# Dose- and Time-Dependent Cytotoxicity of Layered Black Phosphorus in Fibroblastic Cells

**DOI:** 10.3390/nano8060408

**Published:** 2018-06-06

**Authors:** Su-Jin Song, Yong Cheol Shin, Hyun Uk Lee, Bongju Kim, Dong-Wook Han, Dohyung Lim

**Affiliations:** 1 Department of Cogno-Mechatronics Engineering, College of Nanoscience & Nanotechnology, Pusan National University, Busan 46241, Korea; songsj86@gmail.com; 2 Research Center for Energy Convergence Technology, Pusan National University, Busan 46241, Korea; choel15@naver.com; 3 Advanced Nano-surface Research Group, Korea Basic Science Institute (KBSI), Daejeon 34133, Korea; leeho@kbsi.re.kr; 4 Dental Life Science Research Institute, Seoul National University Dental Hospital, Seoul 03080, Korea; bjkim016@gmail.com; 5 Department of Mechanical Engineering, Sejong University, Seoul 05006, Korea

**Keywords:** black phosphorus, 2D nanomaterial, cytotoxicity, biomedical application

## Abstract

Black phosphorus (BP) is a monolayer/multilayer two-dimensional (2D) nanomaterial, which has recently emerged as one of the most attractive 2D nanomaterials due to its fascinating physicochemical and optoelectronical properties. Layered BP may have promising applications in biomedical fields, such as drug delivery, photodynamic/photothermal therapy and bioimaging, although its intrinsic toxicity has not been fully elucidated yet. In the present study, the cytotoxicological effects of layered BP on both cell metabolic activity and membrane integrity were investigated. Layered BPs were prepared using a modified ultrasonication-assisted solution method, and their physicochemical properties were characterized. The dose- and time-dependent cytotoxicity of layered BP was assessed against L-929 fibroblasts. Our findings indicate that the cytotoxicity of BPs is proportionally dependent on their concentration and exposure time, which is affected by the oxidative stress-mediated enzyme activity reduction and membrane disruption. On the other hand, layered BPs did not exhibit significant cytotoxicity at concentrations lower than 4 μg/mL. Therefore, it is suggested that layered BPs can be effectively utilized as therapeutic delivery carriers and imaging agents.

## 1. Introduction

Over the last decade, tremendous research has been conducted to understand and explore the various types of two-dimensional (2D) nanomaterials. This research has found that 2D nanomaterials have a promising potential in a variety of applications, such as optoelectronics, photonics, energy storage and conversion, and biomedicine [1,2]. Among monolayer/multilayer 2D nanomaterials, layered black phosphorus (BP) has recently emerged as an attractive novel one due to its distinct structure, with phosphorenes stacked in several layers via van der Waals forces, and has been acknowledged as one of the most stable allotropes of the phosphorus family [3,4,5,6,7,8]. Some studies have already shown the potential of BP in biomedical applications, such as drug delivery, photodynamic/photothermal therapy and bioimaging [1,9,10,11,12,13]. However, several controversial results regarding the toxicity of BP have been reported, which means that an in-depth understanding of the cytotoxicity and underlying mechanism of BP is of utmost importance.

A series of studies reported that layered BP has little to no toxic effects, which means that it can be employed as a biomedical material [1,6,13]. It has been found that, while the BPs can induce cell apoptosis and necrosis owing to the transient intracellular reactive oxygen species (ROS)-mediated oxidative stress, the induced oxidative stress can be gradually restored to normal levels with no long-term inflammatory reaction or obvious damage to an in vivo mouse model [14]. Moreover, BP nanosheets can be used as drug delivery vehicles because they have pH- or photo-responsive drug release characteristics as well as a high drug loading efficiency [1,9]. Additionally, BP has been found to possess both outstanding near-infrared photothermal performance and photodynamic activity, which allows it to be utilized for photothermal and photodynamic therapy [9,10,11,12,15]. However, although these studies on the biomedical potential of BP could provide valuable guidelines for the essential understanding of the biological effects of BP, the issue of the potential toxicity of BP remains unresolved. In particular, the toxicity of layered BPs is highly varied depending on their concentration, size, shape, surface chemistry, and exposure time, which is similar to the other 2D nanomaterials, such as graphene and its derivatives [14,16,17]. Therefore, prior to the use of layered BP in biomedical applications, it is urgently necessary to investigate its toxicological effects. Hence, in the present study, we assessed the cytotoxicity of layered BP on fibroblastic cells according to its concentration and exposure time, using cytotoxicity assays with different end-points, including the cell metabolic activity, membrane integrity and intracellular ROS production. Our findings revealed that layered BPs showed dose- and time-dependent cytotoxicity, which are caused by oxidative stress-mediated enzyme activity reduction and membrane disruption, but they did not exhibit significant cytotoxicity at a low concentration. These dose- and time-dependent cytotoxicity profiles of layered BPs can be quite informative and useful for their development as biocompatible therapeutic delivery carriers and imaging agents.

## 2. Materials and Methods

### 2.1. Preparation and Characterization of Layered BP

Layered BP was prepared by exfoliation of bulk BP crystals using a modified ultrasonication-assisted solution method, as described elsewhere [13]. Fourier transform infrared (FT-IR) spectroscopy was used to characterize the layered BP. The FT-IR spectrum of layered BP was collected using an FT-IR spectroscope (Nicolet Co., Madison, WI, USA) with a resolution of 4.0 cm^−1^ and 16-times scanning in the wavelength range of 750–4000 cm^−1^. The surface topography of layered BP was analyzed by atomic force microscopy (AFM; NX10, Park Systems Co., Suwon, Korea) in air at room temperature. Imaging was carried out in non-contact mode with a Multi 75 silicon scanning probe at a resonant frequency of ~300 kHz. The average hydrodynamic size of layered BPs was determined using a Zetasizer (Nano ZS, Malvern Instruments, Worcestershire, UK).

### 2.2. In Vitro Assays for Cytotoxicity Evaluation of Layered BP

L-929 fibroblastic cells were routinely cultured in Dulbecco’s modified Eagle’s Medium (DMEM, Welgene, Daegu, Korea) supplemented with 10% fetal bovine serum (Welgene) and 1% antibiotic-antimycotic solution (Sigma-Aldrich Co., Saint Louis, MO, USA) at 37 °C in a humidified atmosphere containing 5% CO_2_. The cell viability of L-929 cells, treated with layered BP for 24 h, 48 h and 72 h, was assessed by a cell counting kit-8 (CCK-8) assay (Dojindo, Kumamoto, Japan) according to the manufacturer’s instructions. Briefly, L-929 fibroblasts were seeded at a density of 1 × 10^4^ cells/mL on a 96-well plate and incubated for 24 h. Subsequently, the cells were treated with various concentrations of layered BP suspended in culture medium (0 to 125 μg/mL) and then incubated with a CCK-8 solution for the last 2 h of the culture period (24 h, 48 h and 72 h) at 37 °C in the dark. The absorbance was measured at 450 nm using an enzyme-linked immunosorbent assay (ELISA) reader (SpectraMax^®^ 340, Molecular Device Co., Sunnyvale, CA, USA). The cell viability was determined to be the percentage ratio of the absorbance values in the cells (incubated with layered BP) to those in untreated control groups (0 μg/mL).

The cell membrane integrity was investigated by monitoring the release of lactate dehydrogenase (LDH) using an LDH assay kit (Takara Bio Inc., Shiga, Japan). After 24 h of incubation with various concentrations of layered BP, the supernatant from each cell culture was transferred to a new 96-well plate. Next, the LDH assay solution was added to each well and then incubated for 30 min at room temperature in the dark. The absorbance was measured at 490 nm using an ELISA reader.

The intracellular ROS production was detected using an ROS assay kit (OxiSelect™; Cell Biolabs, Inc., San Diego, CA, USA). Typically, L-929 cells were plated in a 96-well plate (1 × 10^4^ cells/mL) and incubated for 24 h. The cells were treated with increasing concentrations of layered BP for 24 h. Each cell culture was washed with Dulbecco’s phosphate-buffered saline (DPBS, Gibco, Rockville, MD, USA) and then incubated with 2′,7′-dichloroflurorescein diacetate (DCFH-DA), a cell-permeable fluorogenic probe, for 30 min at 37 °C in the dark. The cells were then imaged using an inverted fluorescence microscope (IX81, Olympus, Melville, NY, USA); the fluorescence intensity was determined by a fluorescence plate reader (VICTOR^3^ Multilabel Counter, PerkinElmer, Inc., Waltham, MA, USA) with excitation and emission wavelengths of 480 nm and 530 nm, respectively. The fluorescence intensity was expressed as the fold-increase over the values of the untreated control groups.

For morphological observations, the time-lapse images of L-929 cells treated with 10 μg/mL of layered BP were acquired every 1 h for 12 h of incubation. The percentage of live cells was estimated by calculating the ratio of the number of attached cells, defined as cells with a spindle-like morphology (i.e., aspect ratio larger than 1) or specialized subcellular structures, such as lamellipodia, filopodia, stress fibers, and membrane protrusions, to the total number of cells [18,19,20,21,22].

### 2.3. Statistical Analysis

All variables were tested in three independent cultures for each experiment, which were repeated twice (*n* = 6). All presented data were expressed as average ± standard deviation. Statistical comparisons were carried out by a one-way analysis of variance (SAS Institute Inc., Cary, NC, USA), followed by a Bonferroni test for multiple comparisons. A value of *p* < 0.05 was considered statistically significant.

## 3. Results and Discussion

### 3.1. Characteristics of Layered BP

**Figure 1:**
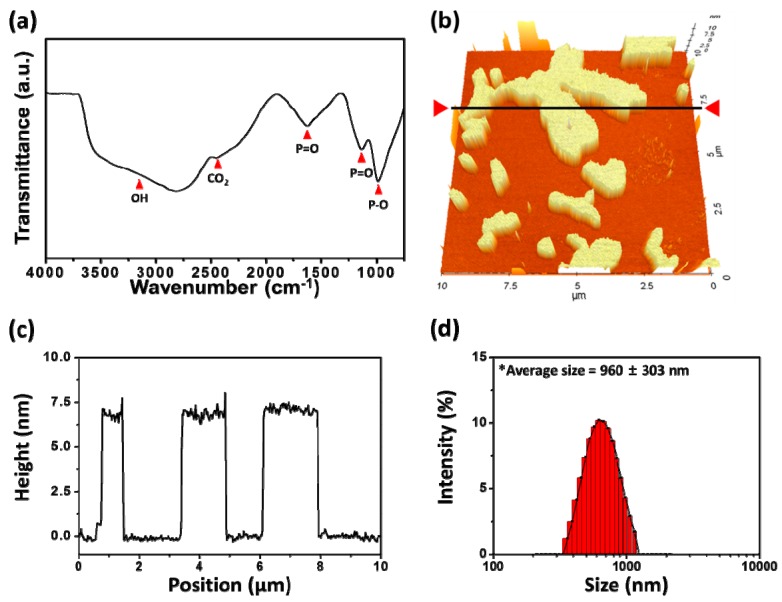
Characterizations of layered BP. (**a**) FT-IR spectrum of layered BP; (**b**) AFM image and (**c**) the height profile of layered BP along the black line marked in (**b**); (**d**) Hydrodynamic size distribution histogram of layered BP.

The physicochemical properties of layered BP were characterized by FT-IR spectroscopy and AFM (Figure 1). The FT-IR spectrum of layered BP showed the characteristic peaks of BP crystals (Figure 1a). A noticeable peak was observed near 1000 cm^−1^, attributed to the stretching vibrations of P–O [23]. The peaks found near 1140 and 1620 cm^−1^ represented the P=O stretching modes of layered BP [23,24]. On the other hand, broad absorption bands were observed, ranging from 2400 cm^−1^ to 3500 cm^−1^, which could be attributed to the CO_2_ stretching and OH stretching vibrations due to exposure of the layered BP to ambient atmosphere. The surface topographic image of layered BP is presented in Figure 1b. Most layered BP were found to have a 2D layer structure, and the average height was about 6.87 ± 0.58 nm (Figure 1b,c). Considering the thickness of the BP monolayer (0.53 nm), the layered BP was composed of several BP monolayers [25]. Moreover, the hydrodynamic size of 2D nanomaterials is of great importance in biomedical applications, because it has a marked effect on the interactions between 2D nanomaterials and cells [17,26,27,28,29]. The hydrodynamic size of the BPs used in the present study was found to be 960 ± 303 nm (Figure 1d).

### 3.2. Dose-Dependent Cytotoxicity of Layered BP

**Figure 2:**
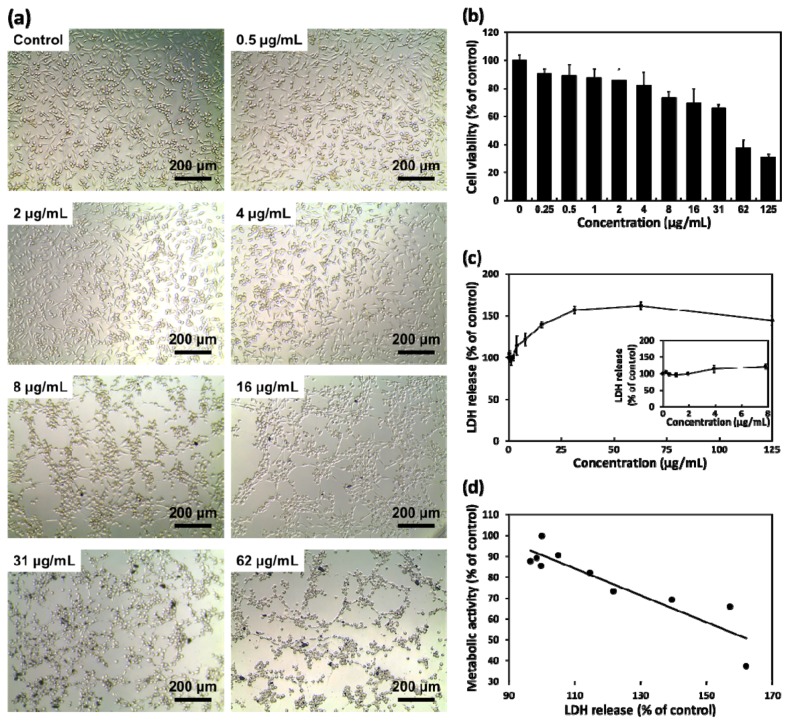
(**a**) Representative optical microscopy images of L-929 fibroblasts cultured with layered BP (0, 0.5, 2, 4, 8, 16, 31 and 62 μg/mL); (**b**) Cell viability and (**c**) LDH release profile of L-929 fibroblasts after 24 h of incubation with various concentrations of layered BP; (**d**) Correlation coefficient plot between metabolic activity and LDH release for cells cultured with layered BP at concentrations ranging from 0 to 62 μg/mL.

To investigate the cytotoxic effects of layered BP on L-929 fibroblasts according to its concentration, cells were treated with increasing concentrations of layered BP (0 to 125 μg/mL) for 24 h, and the morphology of the cells was observed (Figure 2a). There were no significant differences in the number and morphology of L-929 fibroblasts at concentrations of up to 4 μg/mL of layered BP. On the other hand, the cells with aggregated BPs exhibited an abnormal morphology and a significant decrease in cell number at concentrations higher than 8 μg/mL, clearly indicating that layered BPs exhibit dose-dependent cytotoxicity. From the CCK-8 assay, based on the cell metabolic activity (Figure 2b), it was found that the cell viability of L-929 fibroblasts decreased as BP concentration increased. At relatively low concentrations (~4 μg/mL), over 82% of fibroblasts were viable, whereas the cell viability of the control at 62 μg/mL decreased to approximately 37%. These findings are inconsistent with previous reports, which found that BP derivatives, including BP nanosheets and nanodots, were nontoxic to several types of cells even when BP concentration was as high as 1000 μg/mL [1,6,9,13]. These conflicting results may be due to size effects. It was demonstrated that layered BPs show a size-dependent cytotoxicity; larger BPs (with lateral size of ~880 nm) were more cytotoxic than smaller ones (with lateral size of ~210 nm) [17]. As shown in Figure 1d, the average lateral size (~960 ± 303 nm) of layered BPs used in this study was relatively larger than that used in other investigations, which can result in greater toxic effects on cells.

### 3.3. Membrane Disruption and ROS Production Induced by Layered BP

On the other hand, interesting results were found concerning the cytotoxicity of layered BPs. The cytotoxic effects of layered BPs can be ascribed to membrane disruption [17,30]. Therefore, we investigated the cytotoxicity of layered BPs using LDH assays based on the cell membrane integrity (Figure 2c). The extracellular release of LDH has been extensively used for investigating cell membrane integrity, because LDH, a stable cytoplasmic enzyme, can only be released into extracellular fluids upon plasma membrane disruption [31]. As shown in Figure 2c, a significant LDH release was detected at high concentrations of layered BP (≥16 μg/mL). The LDH release increased to approximately 140% of the control at 16 μg/mL of BP, indicating that high concentrations of layered BPs induced a significant membrane disruption. A slight decrease in LDH release, observed at 125 μg/mL, can be due to the decrease in the total cell number. For CCK-8 and LDH assay results, the calculated value of the corresponding correlation coefficient was −0.91 (Figure 2d), implying that the effects of layered BP on cell metabolic activity and membrane integrity were shown to have a high negative correlation.

**Figure 3:**
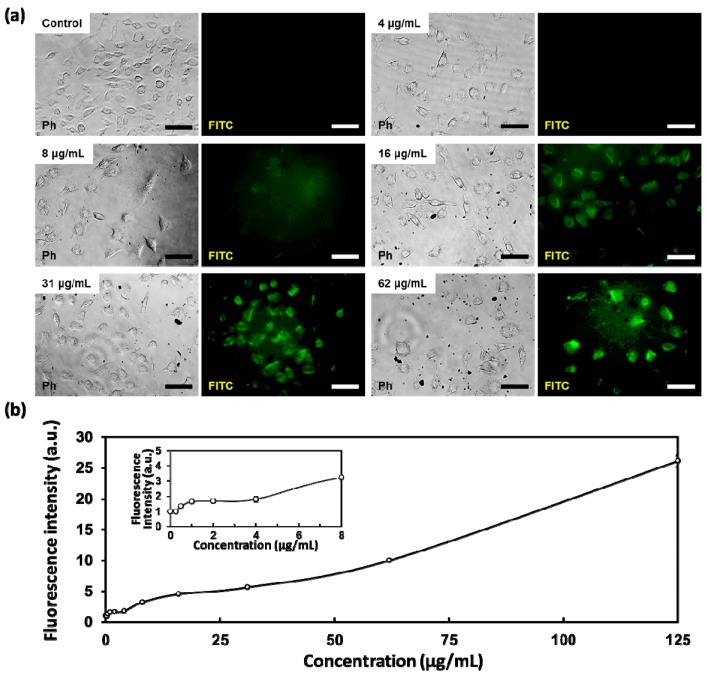
(**a**) Representative fluorescence microscopy images of oxidized DCF fluorescence in L-929 fibroblasts treated with various concentrations of layered BP (0, 4, 8, 16, 31 and 62 μg/mL) for 24 h; and (**b**) quantification of oxidized DCF fluorescence intensity. The scale bars are 100 μm.

At the same time, the dose-dependent cytotoxicity of layered BPs can also be due to oxidative stress. To further investigate the cytotoxicity of layered BPs, the effects of BPs on intracellular ROS generation were evaluated using an ROS-sensitive fluorogenic probe DCFH-DA. The DCFH-DA, a cell-permeable fluorophore, can be readily diffused into cells and subsequently deacetylated by cellular esterases to non-fluorescent DCFH (2′,7′-dichlorodihydrofluorescin). The internalized DCFH is quickly oxidized to highly fluorescent DCF by intracellular ROS. Hence, the intracellular fluorescence of DCF reflects the oxidative stress attributed to the intracellular ROS production. As shown in Figure 3a, the minimal fluorescence was detected at low concentrations of layered BP (≤4 μg/mL), while obvious green fluorescence was detected in L-929 cells after incubation with concentrations of layered BP higher than 8 μg/mL. In addition, the fluorescence intensity was significantly (*p* < 0.05) enhanced with increasing concentrations of layered BPs (Figure 3b). It has been documented that the cytotoxicity of BP nanomaterials causes oxidative stress, such as the reduction of enzyme activity, lipid peroxidation and DNA breaks, caused by intracellular ROS production [14]. Thus, even though the size of the layered BP used in the present study was different from that used in previous studies, the cytotoxicity of layered BPs is proportionally dependent on their concentration, which can be attributed to the reduction of metabolic activity owing to oxidative stress. From our in vitro cytotoxicity assay results with different end-points (the cell metabolic activity, membrane integrity and intracellular ROS production), it was revealed that the dose-dependent cytotoxicity of layered BPs was due to both membrane disruption and oxidative stress-mediated metabolic activity reduction.

### 3.4. Time-Dependent Cytotoxicity of Layered BP

**Figure 4:**
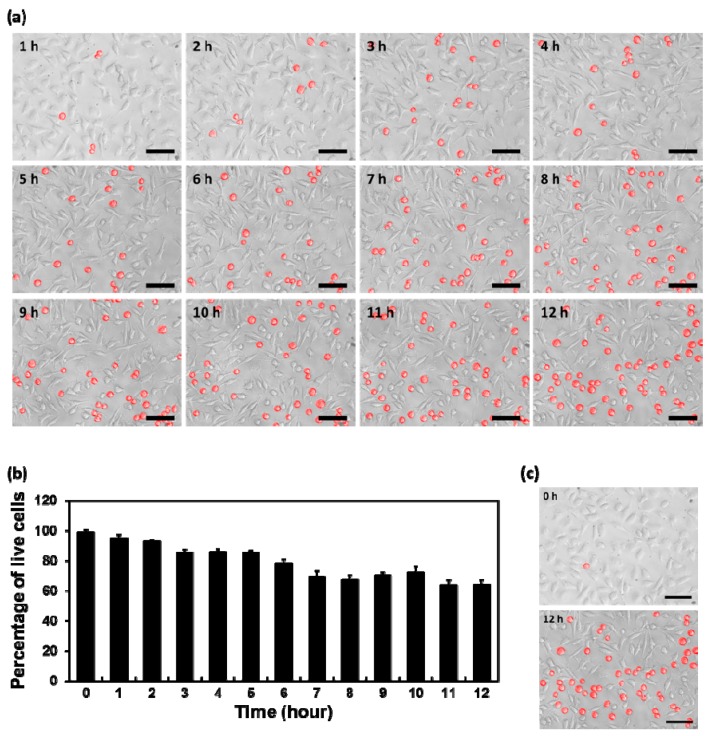
(**a**) Time-lapse images of L-929 cells treated with 10 μg/mL of layered BP for an initial 12 h at an interval of 1 h; (**b**) Quantification of the percentage of live cells for 12 h; (**c**) Optical microscopy images of L-929 fibroblasts treated with 10 μg/mL of layered BP for 0 and 12 h. The scale bars are 100 μm.

To further evaluate the toxic effects of layered BPs on cells, we observed the morphological changes of L-929 fibroblasts and estimated the number of live cells. The time-lapse images of cells, treated with 10 μg/mL of layered BP for an initial 12 h at an interval of 1 h, are shown in Figure 4a. The number of live cells was estimated by quantifying the ratio of the number of attached cells to the total number of cells (Figure 4b). Because adherent cells, including fibroblastic cells, have to be attached to appropriate substrates in order to survive, the cells, which did not show typical fibroblastic morphology, were considered to be dead [18,19,20,21,22]. It was observed that the number of cells with apoptotic morphology (marked in red) increased throughout incubation with layered BPs for the initial 12 h (Figure 4a). In particular, the live cells decreased significantly (*p* < 0.05) after 6 h of incubation with layered BPs (Figure 4b). The morphological changes were clearly observed by comparing optical microscopy images, taken every hour for 12 h (Figure 4c). These results implied that the cytotoxicity of layered BPs is also dependent on their exposure time.

**Figure 5:**
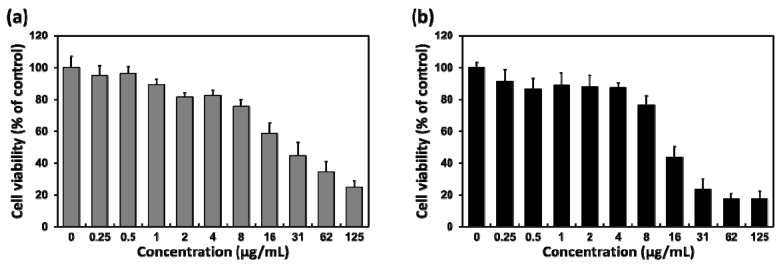
Cell viability profiles of L-929 fibroblasts after (**a**) 48 and (**b**) 72 h of incubation with various concentrations of layered BP.

The cell viability of L-929 fibroblasts, incubated with layered BP for 48 and 72 h, was evaluated to further examine the time-dependent cytotoxicity of layered BP, as shown in Figure 5a,b, respectively. The cytotoxic effects of layered BPs after 48 and 72 h are also dose-dependent, which is similar to the results after 24 h. Additionally, the cell viability after 48 and 72 h decreased more than it did after 24 h, as the incubation time with layered BPs had increased, and the decrease in cell viability after 72 h was more significant than after 48 h. At a concentration of 16 μg/mL, the cell viability after 48 and 72 h decreased to approximately 60% and 45% of the control, respectively. These results indicated that the cytotoxic effects of layered BPs were also dependent on exposure time. Consequently, it was revealed that the layered BP exhibited dose- and time-dependent cytotoxicity, as a result of membrane disruption and oxidative stress-mediated metabolic activity reduction caused by the accumulation of intracellular ROS as well as the interactions between layered BPs and cells. However, it is worth noting that the layered BPs were not significantly cytotoxic at concentrations lower than 4 μg/mL, suggesting that layered BPs in the range of only a few μg/mL can be effectively used in biomedical applications, such as therapeutic delivery carriers and imaging agents. Furthermore, to improve biocompatibility and biological activity, BPs can be conjugated or modified with various functional compounds, such as biocompatible polymers, nanoparticles and drugs [1,10,12,15]. It has been revealed that the encapsulation of BPs with poly(lactic-*co*-glycolic acid), a biodegradable polymer, allows not only the enhancement of biocompatibility, but also the degradation of nontoxic phosphate and phosphonate [12]. These results indicated that, although the cytotoxicity of BPs is closely dependent on their concentration and exposure time, the BPs with the desirable modification can be compatibly employed in biomedical applications, even at concentrations higher than 8 μg/mL. In summary, it is suggested that BP has a promising potential as a biomedical material.

## 4. Conclusions

This study aimed to investigate the dose- and time-dependent cytotoxicity of layered BPs against L-929 fibroblasts. It was revealed that the cytotoxicity of layered BPs was proportionally dependent on their concentration and exposure time. These cytotoxic effects of layered BPs are found to be due to both oxidative stress-mediated enzyme activity reduction and membrane disruption. On the other hand, the cytotoxicity of layered BPs is not significant at concentrations lower than 4 μg/mL. Taken together, this work suggests that layered BPs can be effectively used in biomedical applications, such as therapeutic delivery carriers and imaging agents, although further comprehensive studies are undoubtedly necessary to fundamentally explore and understand the more detailed mechanisms behind the toxic effects of BPs.
